# Visualizing Hot‐Carrier Expansion and Cascaded Transport in WS_2_ by Ultrafast Transient Absorption Microscopy

**DOI:** 10.1002/advs.202105746

**Published:** 2022-02-01

**Authors:** Qirui Liu, Ke Wei, Yuxiang Tang, Zhongjie Xu, Xiang'ai Cheng, Tian Jiang

**Affiliations:** ^1^ College of Advanced Interdisciplinary Studies National University of Defense Technology Changsha 410073 P. R. China; ^2^ State Key Laboratory of High Performance Computing College of Computer National University of Defense Technology Changsha 410073 P. R. China; ^3^ Beijing Institute for Advanced Study National University of Defense Technology Beijing 100000 P. R. China

**Keywords:** exciton diffusion, heat conduction, hot‐carrier expansion, transient absorption microscopy, tungsten disulfide

## Abstract

The competition between different spatiotemporal carrier relaxation determines the carrier harvesting in optoelectronic semiconductors, which can be greatly optimized by utilizing the ultrafast spatial expansion of highly energetic carriers before their energy dissipation via carrier–phonon interactions. Here, the excited‐state dynamics in layered tungsten disulfide (WS_2_) are primarily imaged in the temporal, spatial, and spectral domains by transient absorption microscopy. Ultrafast hot carrier expansion is captured in the first 1.4 ps immediately after photoexcitation, with a mean diffusivity up to 980 cm^2^ s^−1^. This carrier diffusivity then rapidly weakens, reaching a conventional linear spread of 10.5 cm^2^ s^−1^ after 2 ps after the hot carriers cool down to the band edge and form bound excitons. The novel carrier diffusion can be well characterized by a cascaded transport model including 3D thermal transport and thermo‐optical conversion, in which the carrier temperature gradient and lattice thermal transport govern the initial hot carrier expansion and long‐term exciton diffusion rates, respectively. The ultrafast hot carrier expansion breaks the limit of slow exciton diffusion in 2D transition metal dichalcogenides, providing potential guidance for high‐performance applications and thermal management of optoelectronic technology.

## Graphical abstract

A novel spatiotemporal photoexcitation which includes a cascaded process of hot‐carrier expansion, negative contraction, and exciton diffusion is studied in 2D WS_2_, determining an initial average diffusivity of nearly 10^3^ cm^2^ s^−1^ that is 10^2^ times higher than the followed excitonic. This discovery has great potential for improving the efficiency and thermal management of transition metal dichalcogenides‐based optoelectronic devices.

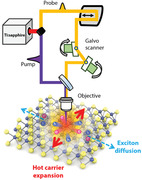

## 1 Introduction

The carrier loss during drift or diffusion constitutes one of the main sources limiting the efficiency of optoelectronic devices.^[^ ^1^, ^2^ ^]^ Before being collected by the electrode, carriers have already lost large amounts of energy through interband recombination or defect trapping, with the energy being released into the environment in the form of photon emission or thermal radiation rather than converted into a photocurrent.^[^ ^2^, ^3^ ^]^ Such carrier loss can be effectively prevented by improving the spatial migration rate via scatter‐free ballistic transport or superdiffusion.^[^ ^4^, ^5^, ^6^, ^7^, ^8^ ^]^ In recent years, 2D transition metal dichalcogenides (TMDCs), deemed potential materials for next‐generation optoelectronics owing to their excellent properties,^[^ ^9^, ^10^, ^11^ ^]^ have been widely used in the fields of field‐effect transistors,^[^ ^9^, ^12^ ^]^ solar cells,^[^ ^13^ ^]^ light‐emitting devices,^[^ ^14^ ^]^ etc. Despite the great success in improving the device efficiency,^[^ ^15^, ^16^, ^17^ ^]^ the underlying photophysics of pristine 2D TMDCs, especially for the real‐space diffusion of nonequilibrium excited‐state carriers, remains unclear.

Intensive research efforts have been spent on studying the exciton transport in 2D TMDCs,^[^ ^18^, ^19^, ^20^, ^21^, ^22^, ^23^, ^24^ ^]^ whose focus lies on timescales larger than picoseconds. At this stage, equilibrium has been established between the carrier and lattice, and the bound, band‐edge exciton dominates the excited states. The reported exciton diffusivity of 2D TMDCs is on the order of 10 cm^2^ s^−1^, either for monolayer or multilayer (multilayer WS_2_ with a thickness slightly thinner than 10 nm presents the sizable electricity due to the avoidance of charge screening from the bulk, meanwhile surface defects or interfacial scattering from the few layer),^[^ ^12^, ^18^, ^19^, ^20^, ^25^, ^26^ ^]^ and far less than that of graphene,^[^ ^27^ ^]^ which is incompatible with the expectations for high‐sensitivity applications.^[^ ^15^, ^28^, ^29^ ^]^ Besides, the short exciton lifetime derived from exciton–exciton annihilation and defect trapping also result in inferior carrier mobility.^[^ ^30^, ^31^, ^32^ ^]^ Among those investigations, the optically excited particles’ diffusion in TMDCs is universally associated with a quasi‐equilibrium state (e.g., excitons) and seems irrelevant to the effect of hot carriers. In fact, when pumping the TMDCs with a nonresonant femtosecond laser, high‐energy excited free carriers are inevitably generated, which is confirmed in spectroscopy.^[^ ^33^, ^34^, ^35^ ^]^ However, it is inconsequent that the relaxation of such hot carriers in the time domain is rarely reflected in spatial motion, suggesting that in addition to excitons, other excitation species that support carrier transport should be explored.

**Figure 1:**
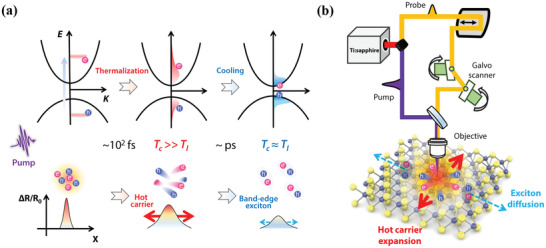
Schematic of photocarrier transport and ultrafast TAM. a) Cartoon of the spatiotemporal carrier dynamics in WS_2_ in momentum (top panel) and real (bottom panel) space. Hot carriers with a high temperature of *T*_c_ are formed within tens of fs via carrier–carrier collision, which drastically expands due to the sharp temperature gradient in the spatial domain. Subsequently, hot carriers cool down and form bound excitons through carrier–phonon scattering in the following ≈1 ps, with the consistent temperature as the lattice *T*_l_. Finally, the long‐term carrier transport is limited by the slow exciton diffusion. b) Schematic of the TAM. The layered WS_2_ is illuminated by an fs pump pulse and then scanned by a weaker probe pulse with the support of galvanometer scanners. The diffusive behaviors of both the hot carriers and excitons are imaged in the form of transient differential reflection Δ*R*/*R*_0_, where the temporal dynamics are obtained by varying the pump‐probe time delay.

Indeed, the earliest photogenerated carriers in 2D TMDCs will experience ultrafast carrier–carrier scattering processes to form quasi‐free hot carriers (the fastest 30 fs for monolayer),^[^ ^34^ ^]^ with a transient carrier temperature (*T*_c_) as high as thousands of Kelvin,^[^ ^35^ ^]^ as shown in **Figure** **1**a. Subsequently, on a timescale of ≈1 ps, the excess energy of the hot carriers is transferred to the lattice via carrier–phonon coupling, leading to a slight rise in the lattice temperature (*T*_l_),^[^ ^34^, ^36^, ^37^ ^]^ accompanied by the formation of bound excitons. In contrast to the “cold‐state” excitons, the hot carriers have a fast velocity, as visualized in silicon,^[^ ^8^ ^]^ leading to transient superdiffusion in the spatial domain. Similar diffusive behaviors have been examined in organic semiconductors and metallic gold,^[^ ^38^, ^39^ ^]^ which may induce anomalous carrier mobility and thus impact the photoresponse and heat dissipation of the devices.

In this work, ultrafast transient absorption microscopy (TAM) is employed to capture the carrier spatiotemporal dynamics of layered tungsten disulfide (WS_2_) with a temporal resolution of ≈400 fs, a timescale approach to the exciton formation here (Figure 1b). Cascaded carrier transport is clearly recorded, which comprises initial ultrafast hot carrier expansion with a diffusivity of nearly 10^3^ cm^2^ s^−1^ (<1.4 ps), negative diffusion, and 100‐fold slower long‐term exciton spread (>2 ps). Treating the motion of particles as macroscopic heat conduction, the transport process is reproduced by a theoretical model including heat conduction equations and corresponding thermo‐optical response.

## 2 Results and Discussion

### 2.1 Hot Carriers and Band‐Edge Excitons in WS_2_

WS_2_ nanoflakes on a Si‐SiO_2_ substrate are prepared by mechanical peeling off from a bulk WS_2_ crystal, and some of them are further transferred onto a Si_3_N_4_‐Ag substrate (see the Experimental Section for more details). Femtosecond carrier dynamics are measured by two types of pump‐probe systems depending on the specific physical parameters. Specifically, the temporal relaxation dynamics of the diverse excitation species are traced by pump‐probe spectroscopy with a 1 kHz repetition frequency, supercontinuum white light probe pulse, while the spatial transport of the carriers is imaged employing TAM with an 80 MHz frequency, quasi‐monochromatic probe pulse working in asynchronous scanning mode (see Supporting Information Note S1 and Figure S1). For both pump‐probe techniques, a nonresonant pumping configuration is used with pump energy of 3.1 eV (400 nm) to inject the initial excited‐state carriers, with pump fluence varying from 8 to 80 µJ cm^−2^. These fluence levels correspond to excitation of hot carrier densities between 1.6 × 10^12^ and 1.6 × 10^13^ cm^−2^, assuming an absorbance of 0.1.^[^ ^40^ ^]^ This density range is regarded as an intermediate regime and does not exceed the Mott limit, i.e., excited states are still in the form of bound excitons after hot carrier cooling, whereas many‐body interactions have already played an important role, and the probe absorption is outside of the linear range.^[^ ^37^, ^41^, ^42^, ^43^ ^]^ Finally, the transient absorption signals are mathematically expressed by the relative reflection: Δ*R*/*R*_0_ = (*R*−*R*_0_)/*R*_0_, where *R*_0_ and *R* are the reflectances before and after pumping. The spatiotemporal resolution of the TAM is shown in Figure S2 in the Supporting Information.

**Figure 2:**
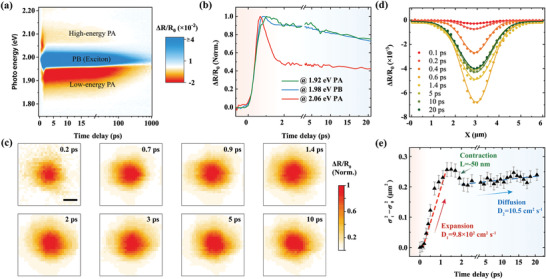
Imaging of spatiotemporal carrier dynamics in WS_2_. a) Typical pseudo‐color Δ*R*/*R*_0_ spectra of layered WS_2_. The photoinduced bleaching (PB) peak is located at the A‐exciton resonance (1.98 eV), with two photoinduced absorption features (PA) on both sides (2.06 and 1.92 eV). b) Normalized temporal dynamics for the three features marked in (a), with fast (slow) component painted in the panel by gradient red (blue). c) 2D Δ*R*/*R*_0_ images of 2.06 eV probe at stepped delay times. All the figures are normalized to highlight the diffusion dynamics. Drastic expansion is observed in the first 1.4 ps. Scale bar: 1 µm. d) Cross‐section 1D profiles of (c) along the *x*‐direction at stepped delay times. Dots are measurement results, lines are Gaussian fits. e) Dynamics of the squared width change *σ_t_* ^2^−*σ*_0_^2^ extracted from the Gaussian fitting of the 1D profile at each delay time. Initial ultrafast hot carrier expansion and long‐term slow exciton diffusion are recorded, with diffusivities of 980 and 10.5 cm^2^ s^−1^, respectively. A tiny negative diffusion can be also observed in the transition region. Dots are experimental results, with error bars reflecting the Gaussian fitting standard deviation, and the red and blue dashed lines are guides corresponding to hot carrier expansion and exciton diffusion, respectively. The pump energy is 3.1 eV, and the fluence is 80 µJ cm^−2^.

With a 3.1 eV and 80 µJ cm^−2^ pump (this condition remains unchanged unless specifically mentioned), **Figure** **2**a shows a typical 2D pseudo‐color transient absorption contour of multilayer WS_2_ near the A‐exciton resonance, where the pump‐probe zero point is set as the starting of the differential signal. Three distinct features can be observed in the spectrum domain, namely, one photoinduced bleaching feature (PB, blue area, a positive signal) at ≈1.98 eV and two photoinduced absorption features (PA, red areas, negative signals) located on both sides of the PB feature (also see Figure S3a, Supporting Information). The PB feature represents the resonant energy of the A‐exciton at the K point of the Brillouin zone of WS_2_, which can be simply ascribed to the band filling effect of those “cold‐state” excitons. We note that the PB feature of the multilayer is slightly redshifted compared to the monolayer feature (see Figure S3b, Supporting Information), which is due to the trade‐off between the reduced exciton binding energy and narrowed quasi‐particle bandgap in thicker WS_2_.^[^ ^44^, ^45^ ^]^

While the PB feature exhibits a clear light–matter interaction, the underlying photophysics for the two PA features is more complicated. As shown in Figure 2a,b, the two PA features exhibit diverse spectral and temporal dynamics. The low‐energy PA feature peaks at 1.92 eV, ≈60 meV lower than the A‐exciton resonance. It shows a biexponential decay dominated by the slow component, coincident with that of the PB feature in the whole measurement window (see Figure S4, Supporting Information), implying that the low‐energy PA feature shares the same excited‐state source as the PB feature (i.e., band‐edge excitons). Except for the early factor of band renormalization,^[^ ^42^ ^]^ such a PA feature may be caused by the formation of biexcitons^[^ ^46^, ^47^, ^48^ ^]^ or trap state excitons^[^ ^32^, ^49^, ^50^ ^]^ induced by pump‐injected band‐edge excitons.

In contrast, the high‐energy PA feature shows distinct characteristics. We attribute the early high‐energy PA feature to the collision broadening effect^[^ ^25^, ^37^, ^51^ ^]^ induced by hot carriers for the following three reasons. First, in the spectral domain, the high‐energy PA appearance is due to the excited‐state absorption caused by the band renormalization or excitonic broadening that shows a quite long high‐energy tail rather than a focused peak, a hallmark of the Fermi–Dirac distribution of the hot carriers.^[^ ^42^, ^52^, ^53^ ^]^ Then, excitonic broadening takes the main role after the hot carrier relaxation thus this PA feature turns to represent the excitons concurrently. Second, in the time domain, the high‐energy PA feature shows a biexponential decay $A_{\mathrm{fast}}e^{-x/\tau_{\mathrm{fast}}}+A_{\mathrm{slow}}e^{-x/\tau_{\mathrm{slow}}}$, with lifetimes of *τ*_fast_ = 0.5 ps and *τ*_slow_ = 120 ps. The fast amplitude has considerable proportion, different from the low‐energy PA and PB features. The fast component is generally believed to be caused by the conversion from the initial hot carriers to band‐edge excitons, which is denoted as intraband scattering.^[^ ^54^, ^55^, ^56^, ^57^ ^]^ In comparison, the slow relaxation component possesses a lifetime approaching that of PB, implying thermal equilibrium for excitons. Third, according to the fluence‐dependent relaxation dynamics, the build‐up lifetime of the high‐energy PA feature decreases with increasing pump fluence (detailed in Figure S5a, Supporting Information), which is caused by the promotion of the collision rate and then the faster carrier thermalization is achieved at a higher pump fluence.^[^ ^33^ ^]^ In sharp contrast, the phonon bottleneck effect at a high pump fluence may severely limit the hot carrier cooling rate and slow down the build‐up of band‐edge excitons (see Figure S5b, Supporting Information).^[^ ^33^, ^58^ ^]^

### 2.2 Hot Carrier‐Dominated Expansion Process in WS_2_

To image the in‐plane diffusion of excited‐state carriers, we employ 2.06 eV probe to perform asynchronous scanning measurements. Figure 2c shows the spatial distributions of the excited states at different probe delays. To highlight the diffusion characteristics, the 2D images are all normalized to the peak amplitude at the corresponding delays. At Δ*t* = 0.2 ps, a timescale within the pulse duration, the initial excitation exhibits a spatial distribution with a full‐width at half‐maximum of ≈1.4 µm, which is controlled by the convolution of the pump and probe pulses. At Δ*t* = 0.7 ps, a noticeable expansion appears and subsequently seems to approach its maximum at Δ*t* = 1.4 ps. Then, the patterns present quite slow linear diffusion within the 20 ps measurement window.

To quantitatively describe such an expansion process originating from hot carriers, we extract the central cross‐section of the patterns at stepped delays, whose results are illustrated in Figure 2d (complete curves are normalized and shown in Figure S6, Supporting Information). Since the in‐plane carrier diffusion in TMDCs is isotropic, the spatial distribution naturally inherits the Gaussian shape of the initial pump and probe pulses. The cross‐section profiles along the *x*‐direction at a delay time of *t* can be expressed as^[^ ^59^ ^]^

(1) $$N\left(x,\;t\right)\;=\;N\left(0,\;t\right)\cdot \exp\left[-\frac{{\left(x-x_{0}\right)}^{2}}{2\sigma_{t}^{2}}\right]$$

where *N* is the carrier density, related to Δ*R*/*R*_0_. *x*_0_ is the center position of the pump pulse and *σ_t_* is the broadened width at different time delays.

Fitting all the 1D profiles with formula (1), *σ_t_* at each delay time can be easily extracted. Then, the average travel distance *L* and diffusion coefficient *D* can be obtained by

(2) $$\left|L\right|=\sqrt{\sigma_{t}^{2}-\sigma_{0}^{2}},\;\;D=\frac{\sigma_{t}^{2}-\sigma_{0}^{2}}{2\Delta t}$$

Figure 2e displays the temporal evolution of the extracted squared width broadening *σ_t_* ^2^−*σ*_0_^2^ within 20 ps probe delay. Ultrafast expansion is found in the initial 1.4 ps, where the hot carriers travel *L* = 500 nm with a diffusivity of *D*_1_ = 980 cm^2^ s^−1^. This expansion also corresponds to the profiles’ uplift in Figure S6 in the Supporting Information. Subsequently, a tiny contraction (represented negatively, *L* = −50 nm) appears at delay times varying from 1.4 to 2 ps, at which time hot carrier cooling and exciton formation have finished. After 2 ps, a linear slow diffusive behavior appears with a much slower diffusivity of *D*_2_ = 10.5 cm^2^ s^−1^, consistent with previous reports of excitons,^[^ ^18^ ^]^ while the initial hot carrier expansion before 1.4 ps in WS_2_ has never been reported to our knowledge. Furthermore, by employing substrate regulation, it is evidenced that the expansion process roots in the real‐space motion of carriers and enhances in a suspended state (see Note S2, Supporting Information). We also investigate the fluence dependence, with results shown in Figure S7 in the Supporting Information. As the pump density decreases, the initial expansion attenuates, while the long‐term diffusion rate remains roughly the same.

Using the same fitting approach, the spatial diffusion dynamics of the band‐edge exciton are also characterized with probe energy of 1.98 eV (see Figure S8a, Supporting Information). A similar but smaller initial expansion (*L* = 310 nm) is captured in the first 1 ps, with an expansion rate of 500 cm^2^ s^−1^, which is followed by a significant contraction (*L* = −150 nm) from 1 to 2 ps. After 2 ps, similar linear, slow diffusion is observed with a diffusivity of 7.3 cm^2^ s^−1^. Such initial exciton expansion is nearly compensated by the subsequent contraction, which is quite different from the trend of 2.06 eV. Additionally, we study the diffusion dynamics of the low‐energy PA feature at 1.92 eV (see Figure S8b, Supporting Information). An opposite diffusive behavior is found for this low‐energy feature, i.e., an initial sharp contraction instead of expansion in the first 3 ps, with a rate of −240 cm^2^ s^−1^. Then, an analogous slow diffusion of 9.5 cm^2^ s^−1^ is obtained after 4 ps. The inconsistent and complex diffusive behaviors of the PB and low‐energy PA features are discussed later and in Note S4 in the Supporting Information. Here, we focus primarily on the spatial diffusion dynamics of the hot carriers, namely, the early expansion of 2.06 eV, which can be theoretically described with the cascaded transport model discussed below.

### 2.3 Hot Carrier‐Exciton Cascaded Transport Model

The two‐stage diffusion of hot carriers can be simply attributed to the combined effects of the carrier temperature‐induced pressure gradient (the initial fast expansion) and phonon‐limited diffusion (long‐term linear diffusion), similar to the case of silicon semiconductors.^[^ ^8^ ^]^ However, this qualitative model cannot elucidate the transition region between the two diffusion stages, i.e., the contraction region at a time delay of 1.4–2 ps (Figure 2e). We note that recently, Block et al.^[^ ^39^ ^]^ reported hot‐electron diffusion in thin gold films, which presents almost the same diffusive dynamics as in our work, including the subtle contraction in the transition regime. Block et al. found that such complex diffusion can be accurately modeled by a 3D space model combining spatial thermal transport and a thermo‐optical response. Inspired by this, we speculate that the hot carriers in TMDCs can be described by a similar macroscopic model, which also helps avoid the nonlinear relationship between the pump‐induced carrier population and optical response, affected by the saturation effect^[^ ^4^ ^]^ (see Figure S9, Supporting Information). However, note that the conditions are dissimilar between gold and TMDCs. First, gold is a metal whose excited states are dominated by free carriers, while both hot carriers (ultrafast timescale) and bound excitons (long timescale) govern the excitation of TMDCs. This implies that the thermo‐optical response of gold film can be simply described by a Drude model, which is not suitable for the case of TMDCs.^[^ ^31^, ^60^ ^]^ Second, diffusion mismatch is expected in the transition region due to the distinct diffusivities of the hot carriers and excitons, which are absent in the gold film.

**Figure 3:**
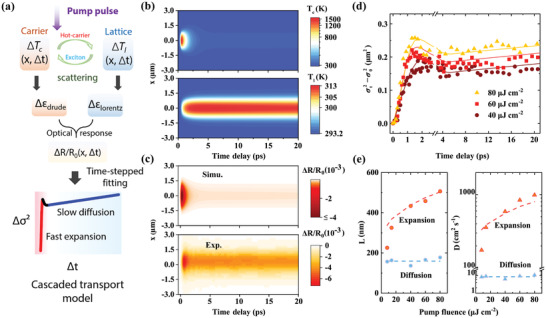
Theoretical modeling of cascaded carrier transport. a) Schematic of the hot carrier‐exciton cascaded transport model. The pump‐induced complex permittivity change ∆*ε* stems from two subsystems: carrier temperature‐limited hot carrier expansion and lattice thermal transport‐limited exciton diffusion, which can be characterized by Drude–Smith and Lorentz models, respectively. Then, ∆*ε* is converted to Δ*R*/*R*_0_ through the Fresnel formula, and ∆*σ* ^2^ is extracted by Gaussian fitting of the spatial profiles at each delay time. b) Calculated spatiotemporal map of the carrier (top) and lattice (bottom) temperatures. c) Calculated (top) and experimental (bottom) spatiotemporal maps of Δ*R*/*R*_0_. d) Evolution of the squared width change at different pump fluences. Dots (lines) are experimental (calculated) results. Ultrafast expansion and slow exciton‐limited diffusion can be observed in the first 1.4 ps and after 2 ps, respectively. e) Extracted diffusion length (left) and diffusivity (right) in both diffusive regions at increasing pump fluence. Dots (dashed lines) are experimental (calculated) results. The fast expansion speeds up with increasing fluence, while the slow exciton‐limited diffusion roughly remains constant. The pump fluence is 80 µJ cm^−2^ for (b) and (c).

Taking the differences into account, we establish a cascaded transport model involving both hot carrier‐dominated expansion and exciton‐limited diffusion, as shown in **Figure** **3**a. Briefly, upon nonresonant pumping, hot carriers with high excess energy are initially injected after ultrafast carrier–carrier scattering. This high‐temperature degenerate gas exhibits a Fermi–Dirac energy distribution^[^ ^42^ ^]^ and a transient free‐carrier response,^[^ ^60^ ^]^ suggesting a similar treatment as for noble metals.^[^ ^39^, ^61^ ^]^ Thus, the optical permittivity change induced by the hot carriers can be well described by the Drude–Smith model ∆*ε*_drude_.^[^ ^31^, ^50^ ^]^ Then, the excess energy is transferred to the lattice via carrier–phonon scattering (≈1 ps), leading to cooling of the hot carriers and formation of band‐edge excitons. The optical permittivity change caused by excitons is characterized by the Lorentz oscillator model ∆*ε*_lorentz_.^[^ ^54^, ^62^, ^63^ ^]^ As the carriers relax in the time domain, they simultaneously diffuse in the space domain, well described using a 3D thermal transport model with the dielectric response. Finally, by considering these joint parts in both the time and space domains, the whole permittivity and thus the reflectivity change of WS_2_ at different time delays and spatial locations can be calculated using the Fresnel formula.

First, assuming the cooled excitons are at equal temperature as the lattice, we consider the spatiotemporal relaxation of the temperatures of the hot carrier (*T*_c_(***r***, *t*)) and band‐edge exciton or lattice (*T*_l_(***r***, *t*)). In Cartesian coordinate, they can be calculated using the following 3D thermal transport equations and boundary conditions^[^ ^39^, ^61^ ^]^

(3a) $$\begin{array}{ccc} C_{c}\;\frac{\partial T_{c}}{\partial t} & = & \nabla \cdot \left(\kappa_{c}\cdot \nabla T_{c}\right)-G\left(T_{c}-T_{l}\right)+S,\;\;k_{\mathrm{cz}}\;\frac{\partial T_{c}}{\partial z}{|}_{z=10\;\;\mathrm{nm}}\\ & = & -S_{\mathrm{cs}} \end{array}$$

(3b) $$\begin{array}{ccc} C_{l}\;\frac{\partial T_{l}}{\partial t} & = & \;\nabla \cdot \left(\kappa_{l}\cdot \nabla T_{l}\right)+G\left(T_{c}-T_{l}\right),\;\;\;k_{\mathrm{lz}}\frac{\partial T_{l}}{\partial z}{|}_{z=10\;\;\mathrm{nm}}\\ & = & -S_{\mathrm{ls}} \end{array}$$

(3c) $$C_{s}\;\frac{\partial T_{s}}{\partial t}=\nabla \cdot \left(k_{s}\cdot \nabla T_{s}\right),\;\;k_{s}\frac{\partial T_{s}}{\partial z}{|}_{z=10\;\;\mathrm{nm}}=S_{\mathrm{cs}}+S_{\mathrm{ls}}$$

where *C*, ***κ*** (*k*), and *T* are the heat capacity, thermal conductivity, and temperature, with the subscripts c, l, and s corresponding to the WS_2_ carriers, WS_2_ lattice, and substrate, respectively. Since the thermal conductivity is anisotropic in WS_2_,^[^ ^64^ ^]^ it can be expressed as a diagonal tensor $\kappa_{c(l)}=\left(\begin{array}{c} k_{c(l)}\\ k_{c(l)}\\ k_{c(l)z} \end{array}\right)$ , where *k*_c(l)_ and *k*_c(l)z_ are the in‐plane and through‐plane thermal conductivities. The source *S*(***r***, *t*) represents the initial heat source in WS_2_ originated from the pump pulse, determining the total heat energy of the system, *G* is the carrier–phonon coupling coefficient, *S*_cs_ (*S*_ls_) characterizes the thermal transport from the carriers (lattice) to the substrate at the WS_2_‐substrate interface, and *z* = 10 nm is the WS_2_ thickness (all relevant parameters are detailed in Note S3a, Supporting Information).

Figure 3b shows the typical simulated results for both *T*_c_(*x*, *t*) and *T*_l_(*x*, *t*) (*y* = *z* = 0) under the same pump fluence of 80 µJ cm^−2^, with the corresponding experimental results of Figure 2d. More details about the temperature evolution at the pump center under different pump fluences can be found in Figure S10 in the Supporting Information. Immediately after excitation, the carrier temperature at the pump core quickly rises above 1500 K in 0.6 ps, accompanied by isotropic thermal expansion in the in‐plane spatial domain due to the very large temperature gradient.^[^ ^8^ ^]^ This heat conduction is the root cause of the observed hot carrier expansion discussed above and can be efficiently tuned through the pump fluence (see Figure S7, Supporting Information). After 0.6 ps, carrier–phonon coupling leads to a fast decay of the carrier temperature and simultaneously a slight elevation of the lattice temperature of ≈20 K. Meanwhile, thermal equilibrium is established within 4 ps between the carriers and lattice. After that, both the carrier and lattice temperatures slowly recover to room temperature due to in‐plane thermal transport and substrate leakage.^[^ ^65^ ^]^

Next, for the thermo‐optical response, we consider the contributions of both hot carriers and excitons. The complex permittivity can thus be described by a Drude–Smith–Lorentz model^[^ ^31^ ^]^

(4) $$\begin{array}{ccc} \varepsilon \; & = & \varepsilon_{\infty }\;+\frac{f_{\mathrm{ex}}}{E_{\mathrm{ex}}^{2}-E_{\mathrm{probe}}^{2}-i\gamma E_{\mathrm{probe}}}\\ & & +\frac{iD_{0}}{\omega_{\mathrm{probe}}\left(1-i\omega_{\mathrm{probe}}\tau \right)\left(1-\frac{C}{1-i\omega_{\mathrm{probe}}\tau }\right)} \end{array}$$

where the three terms on the right‐hand side represent the high‐frequency limit, Lorentz component, and Drude–Smith component of the permittivity. More details are provided in Note S3b in the Supporting Information. *E*_ex_ and *f*_ex_ are the resonant energy and oscillator strength of the A‐exciton, *E*(*ω*)_probe_ is the probe energy (angular frequency), and *γ* is the excitonic broadening, which scales linearly with the lattice temperature.^[^ ^37^, ^66^ ^]^ *D*_0_ represents the Drude weight, and *D*_0_/ *f*_ex_ is proportional to the population ratio between hot carriers and excitons, which is governed by the carrier temperature assuming a quasi‐equilibrium between the two excited states.^[^ ^67^ ^]^ The coefficient *C*(0 ≤ *C* ≤ 1) determines the degree of carrier localization,^[^ ^31^ ^]^ and *τ* is the carrier thermalization time related to the carrier temperature.^[^ ^33^ ^]^ A typical calculation of the dynamics of the complex permittivity change is shown in Figure S11 in the Supporting Information, in which the individual contributions of hot carriers (Drude–Smith component) and excitons (Lorentz component) are also noted. As expected, hot carriers dominate the permittivity change in the first 1 ps, while excitons govern them afterward.

Given the complex permittivity change, Δ*R*/*R*_0_ can be simply calculated by the Fresnel formula (see Note S3c, Supporting Information), and the results are provided in Figure 3c for a comparison between the measured and simulated values in both the space and time domains. The same basic trend is observed in the dynamics of the two Δ*R*/*R*_0_ profiles, which initially exhibit a prominent bump, followed by very little broadening for the long‐term evolution. We note that the simulation predicts a larger amplitude drop in the transition region compared to the experimental results. The same phenomenon is also found in gold films, which may be caused by incomplete estimation of the absorbance^[^ ^39^ ^]^ or the Lorentz component. Here, the change of magnitude and linewidth of Δ*R*/*R*_0_ are absolute and relative, respectively, where the linewidth calculation greatly simplifies the trivial factors of absolute values but retains the key spatial matters. Therefore, this deviation in our model does not affect the simulation of the diffusive dynamics.

To further quantitatively analyze the hot carrier diffusion, we apply Gaussian fitting to the simulated Δ*R*/*R*_0_ profile at different probe delays and extract the changes in the squared width broadening, ∆*σ* ^2^ = *σ_t_* ^2^−*σ*_0_^2^. The comparison between these results and the experimental results is shown in Figure 3d. Excellent agreement is found for the various pump fluences, even for the tiny negative diffusion in the transition region. Figure 3e summarizes the comparison of the extracted diffusion length $L\;=\sqrt{\Delta \sigma^{2}}\;$ (left panel) between the hot carriers and band‐edge excitons, as well as their difference in diffusivity *D* (right panel). With increasing pump fluence, the maximum carrier temperature increases, and both the *L* and *D* of the hot carriers correspondingly increase. As the pump fluence increases from 8 to 80 µJ cm^−2^, the expansion length is promoted from 220 nm to more than 500 nm, corresponding to a faster mean expansion rate, from 175 to 980 cm^2^ s^−1^. A deviation is observed between the experimental and simulation values at low power in Figure 3e, which is considered as the measurement loss from the insufficient signal‐to‐noise ratio. In contrast, the diffusion of the band‐edge excitons remains almost unchanged under increasing power due to the slight lattice temperature elevation, as well as the temperature‐insensitive lattice heat capacity and thermal conductance of WS_2_.^[^ ^54^ ^]^

### 2.4 Understanding of Cascaded Carrier Transport

The cascaded carrier transport can be simply understood by the following two limiting cases. In the first 1.4 ps, carrier transport is dominated by hot carriers, exhibiting a sharp temperature gradient and thus a strong heat‐transfer ability. The instantaneous rate of expansion is estimated as *D*_1_ = *k*_c_/*C*_c_, which gradually decays, based on which we obtain the average value of ≈10^3^ cm^2^ s^−1^. After 2 ps, thermal equilibrium is established between the hot carriers and excitons, and carrier transport is limited by the lattice, as the diffusion of band‐edge excitons can be defined as *D*_2_ = (*k*_l_ + *k*_c_)/(*C*_c_ + *C*_l_) after the expansive process. This value is consistent with the general 10 cm^2^ s^−1^.

**Figure 4:**
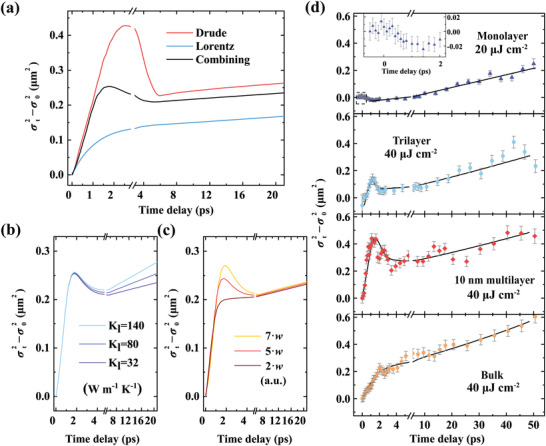
In‐depth discussion of cascaded carrier transport. a) Calculated squared width change obtained from individual Drude–Smith and Lorentz terms, as well as their combination. b,c) Calculated evolutions of the squared width with long‐term diffusion and fast expansion modulated by increasing b) lattice thermal conductance *k*_l_ and c) Drude weight *w*, respectively. d) Carrier transport in WS_2_ with various thicknesses probed at 2.06 eV. Dots are experimental results, and solid lines are guidelines. A zoom‐in of the first 2 ps is shown in the inset for monolayer WS_2_. Both the early nonlinear process and long‐term linear exciton diffusion can be found at all thicknesses. The pump fluence is 80 µJ cm^−2^ for (a)–(c) and marked in (d).

The two‐state carrier diffusion, as well as the abnormal contraction in the transition region, can be profoundly understood by disentangling the hot carrier (Drude–Smith) and exciton (Lorentz) contributions to *σ_t_* ^2^−*σ*_0_^2^, as shown in **Figure** **4**a. Unambiguously, the carrier diffusion is dominated by the ultrafast expansion of hot carriers at the initial, with an expansion rate related to the carrier temperature. Note that on this timescale, the excitonic component also exhibits ultrafast expansion, which is however caused by the exciton formation from expansionary hot carriers instead of the intrinsic exciton diffusion. The long‐term carrier diffusion after 2 ps is limited by the slow exciton diffusion component, at which time hot carriers (free carriers) also exhibit slow diffusion due to the thermal equilibrium between free carriers and excitons. As expected, this long‐term diffusivity scales linearly with *k*_l_ for a constant *C*_l_,^[^ ^68^ ^]^ as shown in Figure 4b. Between the two diffusion regions, the abnormal contraction is caused by the mismatch of the diffusivities of hot carriers and excitons. The contraction amplitude depends on various complex factors, such as the coefficient of the relative ratio *w* between the Drude–Smith and Lorentz components (*w* **∝** *D*_0_/*f*_ex_, see Figure 4c and Note S3b, Supporting Information), the carrier thermal conduction parameters (*k*_c_ and *C*_c_, see Figure S12a,b, Supporting Information), and the carrier temperature‐related carrier–carrier scattering rate (*τ* **∝** *T*_c_^−^ *^*α*^*, see Figure S12c, Supporting Information).^[^ ^33^ ^]^ Generally, a larger contribution of hot carriers to ∆*ε* or enhancement of the hot carrier expansion rate can lead to a more pronounced contraction.

We finally explore the thickness dependence of cascaded transport phenomena in WS_2_, and the results are vividly shown in Figure 4d. In the measurement, the probe wavelength is slightly blue‐shifted (between 2.06 and 2.10 eV) with the decrease of WS_2_ thickness to match the spectral high‐energy PA of different samples. The initial nonlinear behavior and long‐term slow diffusion are universal in all samples. Nevertheless, some differences exist in the expansion and contraction process. The hot carrier expansion gradually diminishes as the WS_2_ thickness decreases and tends to become invisible at the monolayer limit. This weakening has nothing related to the reduced pump power (down to 20 µJ cm^−2^ due to the damage threshold) because the thicker WS_2_ also has obvious expansion under the same power. It may arise from the enhanced quantum confinement^[^ ^44^ ^]^ and then a lower hot carrier contribution in thinner WS_2_. In contrast, the contraction in the transition region becomes more conspicuous in thinner layers. Specifically, the contraction–expansion ratio is near zero in the bulk and increases to about 1/3 and 1/2 in 10 nm and trilayer WS_2_, while it is even greater than 1 in the monolayer. Such thickness‐dependent contraction may stem from the alteration of the carrier–carrier interaction. Carriers in a thinner layer WS_2_ experience a reduced dielectric screening and thus an increased carrier–carrier scattering rate,^[^ ^44^, ^69^ ^]^ leading to a more salient diffusion contraction according to Figure S12c in the Supporting Information. Explicitly, carrier transport depends on the joint interaction of the dielectric environment and particle scattering,^[^ ^70^ ^]^ corresponding to the discussion here. Notably, with a lower pump fluence of 40 µJ cm^−2^ here, the expansion distance of the 10 nm multilayer is even larger than that in Figure 2e, with an average speed over 1700 cm^2^ s^−1^. We attribute this to the high crystal quality without substrate transfer. For photoelectric applications that care about practical efficiency, the performance of multilayer WS_2_ is often superior to the monolayer.^[^ ^12^ ^]^ Therefore, based on the above considerations, this work concentrates mainly on the multilayers.

More interesting features can be investigated in the transition region presented by the negative carrier diffusion, which is attributed to the mismatch between the diffusivities of hot carriers and excitons. Hot carriers expand much faster than excitonic diffusion in the first 1.4 ps, during which they cool down to the band edge to form bound excitons. Consequently, substantial excitons tend to locate at the pump center, while partial hot carriers disperse peripherally (see Note S4 for details, Supporting Information). Thus, carrier contraction naturally occurs when the time delay is scanned from the hot carrier‐dominated to exciton‐dominated region. The contraction deviation between the 2.06 eV probe and 1.98 eV probe may be caused by the defect trapping of polar carrier (electron or hole) during the expansion of the hot carriers (WS_2_ is considered to be slightly *n*‐doped due to sulfur vacancy, which may capture holes during expansion). As a result, the remaining free carriers can qualitatively retain the broadening of the hot carriers but not the band‐edge excitons. An alternative explanation to the contraction deviation is the different mechanisms for PB and PA signals. Specifically, the PB is directly governed by the photogenerated species (both hot carriers and excitons), while the high‐energy PA is generated by the broadened excitonic absorption induced by hot carrier scattering and exciton–exciton scattering. Exhaustive origins may be more than that in our discussions and remain elusive in certain. Nevertheless, the initial hot‐carrier expansion provides a ladder for excitons’ subsequent diffusion, where the expansive speed determines the ladder's length. These two processes jointly determine the effective travel distance of excitons. More systematic studies on the complicated carrier diffusion in different TMDCs and other 2D semiconductors are urgently required in the future.

## 3 Conclusion

In conclusion, we have comprehensively investigated the cascaded carrier transport in layered WS_2_ by ultrafast TAM, which comprises initial ultrafast hot carrier expansion, transitional negative diffusion, and linear exciton spread. Specifically, the fast expansion covers a long‐range distance of 500 nm in 1.4 ps, with a transient speed much higher than the following exciton diffusion. The entire diffusion scenario can be well described by a cascaded transport model, where the pump‐induced complex permittivity change is divided into the Drude–Smith hot carrier term and Lorentz band‐edge exciton term. These results shed new light on the nonequilibrium photophysics in TMDCs, while the cascaded transport contains a superdiffusion that may furnish the feasibility for controlling the heat exchange between carriers and phonons. The fast expansion associated with the excess energy of hot carriers is latent for ultrahigh carrier mobility and even surmounting the limit of efficiency, further offering wide applications in the field of magnetization, thermoelectric, and plasma. Nevertheless, further explanation of the carrier transport is still hindered by the time‐space accuracy of the experimental system, through the advancements of which more in‐depth and probable revolutionary research can be available.

## 4 Experimental Section

### Sample Preparation and Characterization

WS_2_ nanoflakes were mechanically peeled off from bulk WS_2_ crystal onto Si/SiO_2_ substrate. The WS_2_ thickness and morphology were roughly identified by optical contrast and further confirmed by atomic force microscopy (AFM, Innova, German Bruker Corporation) and home‐build TAM (synchronous scanning mode), as shown in Figure S13 in the Supporting Information. A part of layered WS_2_ was further transferred to a partially etched Si_3_N_4_‐Ag substrate by a polymer‐assisted method, which provided a substrate regulation for carrier transport (see Note S2 and Figure S14, Supporting Information) and improved the probe reflectance thus signal–to–noise ratio of the Δ*R*/*R*_0_.

### Femtosecond Pump‐Probe Spectroscopy

A femtosecond pulse from the amplification level of Ti:sapphire laser (Spectra‐Physics, 800 nm central wavelength, 1 kHz repetition rate) was separated into two unequal parts, with the stronger one passing through an optical parametric amplifier (TOPAS) for wavelength tuning, acting as the pump pulse. While the weaker one was focused onto a sapphire crystal to yield a continuous white light (from 470 to 1100 nm), serving as the probe pulse, whose optical path was controlled by the optical delay line. To eliminate common‐mode noise, a portion of the broadband beam was collected directly by optical fiber coupling, taken as the reference. The pump and probe pulses were focused onto the sample through an objective lens (Olympus 20x, 0.4 NA), with a laser spot size of ≈4 µm. Then probe reflected pulse was also collected by another optical fiber and further delivered to a multichannel spectrometer (0.1 nm resolution) with the reference. Finally, the whole spectra were recorded by a CCD camera. The synchronization of the signal collection was achieved by the internal chopper/computer clock, and the transient dynamics was obtained by stepping the delay line at the probe path.

### Spatiotemporal Transient Absorption Microscopy

The TAM was based on the femtosecond pulse from the oscillatory level of the Ti:sapphire laser (Spectra‐Physics, 800 nm central wavelength, 80 MHz repetition rate), as shown in Figure S1 in the Supporting Information. Similarly, the 100 fs laser pulse was divided into two parts, with the main one first modulating by an acousto‐optic modulator (AOM, 300 kHz) and then focusing on *β*‐barium borate (BBO) crystal to double the photon energy, which served as the pump. While the weaker beam was first coupled to a photonic crystal fiber to generate a white light supercontinuum, and then a selected wavelength was filtered out through a narrow band‐pass filter (Thorlabs, 10 nm FWHM), working as the probe. Temporal and spatial dynamics were acquired by stepping the delay line and 2D galvo mirrors (Thorlabs, GVS012) placed on the probe path. After focusing the pump and probe beams onto the sample with a high‐power microscope objective (Olympus 50x, 0.85 NA). Through a monochromator (Sofn Instruments 7ISW30, 0.4 nm spectral resolution), the indicated resonance of probe reflection was filtered and then detected by an avalanche photodetector (Thorlabs, APD120A/M) followed by a dual‐phase lock‐in amplifier (Sine Scientific Instrument, OE1022D). Notably, although the repetition rates of the laser source that might affect the steady‐state temperature and energy shift distinguish spatiotemporal TAM from the transient spectra measurement, the PA and PB signals could be judged by positive and negative values in lock‐in amplifier, respectively. The 2D imaging of Δ*R*/*R*_0_ could be performed using either synchronous or asynchronous scanning modes (see Supporting Information Note S1 for detail).

## Conflict of Interest

The authors declare no conflict of interest.

## Supporting information

Supporting Information

## Data Availability

The data that support the findings of this study are available from the corresponding author upon reasonable request.
