# Supplementary material for: Visualizing Hot‐Carrier Expansion and Cascaded Transport in WS2 by Ultrafast Transient Absorption Microscopy
Source: Adv Sci (Weinh). 2022 Feb 1;9(10):2105746. doi: 10.1002/advs.202105746 (PMC8981895; doi:10.1002/advs.202105746)
Supplement: Supplementary file 1 — Supporting Information [file ADVS-9-2105746-s001.pdf]

WILEY-VCH

ADVANCED SCIENCE
Open Access

Supporting Information

for *Adv. Sci.*, DOI: 10.1002/advs.202105746

Visualizing hot-carrier expansion and cascaded transport in WS_2 by ultrafast transient absorption microscopy

*Qirui Liu, Ke Wei, Yuxiang Tang, Zhongjie Xu, Xiang'ai Cheng and Tian Jiang**

Supporting Information

Visualizing hot-carrier expansion and cascaded transport in WS_2 by ultrafast transient absorption microscopy

*Qirui Liu, Ke Wei, Yuxiang Tang, Zhongjie Xu, Xiang'ai Cheng and Tian Jiang**

Note S1. Measurement details of transient absorption microscopy

Equipped with the space scanning capability, the spatiotemporal TAM setup provides three main functions: (1) Temporal dynamics measurement through the stepped delay line. (2) 2D synchronous imaging of the sample morphology realized by moving motor-driven micropositioning sample stage, during which the pump and probe remain completely overlapped. (3) 2D asynchronous imaging of carrier spatial distribution by scanning the probe around the center of the pump beam, with the help of 2D galvo mirrors. The carrier density N in one specific in-plane spatial direction, say x direction, is represented as[1]

$$N(x,t)=N(0,t)\cdot\exp\left[-\frac{(x-x_0)^2}{2\sigma_t^2}\right] \tag{1}$$

where $\sigma_t=\frac{\mathrm{FWHM}}{2\mathrm{sqrt}(\ln(4))}$ represents a defined profile width that is proportional to the full-width-at-half-maximum (FWHM), and x_0 is the center position of the pump beam. Notably, σ_t depends on the initial pump and probe beams, and the temporal evolution of the carrier distribution, namely, $\sigma_t^2=\sigma_{\mathrm{pump}}^2+\sigma_{\mathrm{probe}}^2+\sigma_{\mathrm{carrier}}^2$. The carrier diffusion length over a period from t_0 to t can be expressed as $|L|=\sqrt{\sigma_t^2-\sigma_0^2}$, and the mean diffusion coefficient is provided as $D=(\sigma_t^2-\sigma_0^2)/2(t-t_0)$. Since σ_{pump} and σ_{probe} do not vary with time, both the L and D depend only on spatial transport. In fact, the real travel distance of carriers will be larger than the measured value, because the spatial behavior within the pulse duration exceeds

the time resolution. Nevertheless, the minimum measurable distance is primarily decided by measurement accuracy (~10 nm) rather than spatial resolution. This accuracy brings the error bar that mainly depends on signal-to-noise ratio and can be calculated by transfer function as

$$\Delta L = 2\sqrt{\sigma_0^2 \cdot (\Delta\sigma_0)^2 + \sigma_t^2 + (\Delta\sigma_t)^2} \quad (2)$$

Note S2. Regulation of carrier transport induced by substrate.

As mentioned above, we have observed the phenomenon of cascade carrier transport in WS_2 experimentally and verified its universality in different thicknesses. However, the hot-carrier expansion does not necessarily come from real-space diffusion. It may be caused by the in-plane relaxation rate difference (annihilation or Auger recombination) brought by the Gaussian distributed pump spot, especially within the temporal resolution. Specifically, considering the carrier concentration in the spot periphery is smaller than the center, where the carrier recombination is faster due to those higher-order processes, the carrier linewidth should increase experimentally. To exclude this possible but important origin, we reproduce the spatial pump-probe measurement on an etched Si_3N_4 substrate under the same WS_2. As shown in FigureS13 a, b, the grating period is 400 nm and the suspension ratio is close to 40%. Therefore, WS_2 is semi-suspended in the etched region. Because of the 1D structure, the substrate effect on the dielectric properties of WS_2 is completely different in the vertical and horizontal directions. If the expansion is caused by real-space carrier transport, the different dielectric properties will inevitably lead to anisotropy. Or if the root cause is relaxation rate difference, the change of substrate will make a little effect.

2D pump-probe imaging was performed with a 3.1 eV pump (80 μJ cm^{-2}) and 2.06 eV probe, as shown in FigureS14a. To highlight the spatial change, the images at different time delays are all normalized. Comparing the results of the uniform substrate (upper) and grating substrate (lower), it can be found that the introduction of the grating causes strong anisotropic transport, and the carrier expansion rate along the grating's horizontal is significantly faster

than that in the vertical direction. This leads the carrier shape from an approximate circle (0.2 ps) to ellipse (0.7 ps), nevertheless, the carrier shapes of bare WS_2 keep circular at every delay. Since the grating gap (400 nm) is less than half of the spot size (~ 1.3 um), the non-uniformity of the whole structure can be ignored for the focused spot (also see morphology imaging of Figure S13d). Moreover, the energy position of A-exciton on the etched substrate is almost equal to that on the flat substrate (see Figure S13c), thus the strain effect caused by the grating can also be ignored. Therefore, the anisotropy is entirely due to the dielectric regulation of the grating. In the horizontal, the carrier transport proceeds smoothly in the semi-suspended WS_2 while it periodically experiences Si_3N_4 and air substrate in the vertical. It can be inferred that the substrate dielectric regulation hinders carrier transport.

Also, for a quantitative description, the carrier transport parallel and perpendicular to the grating are further obtained by 1D asynchronous scanning, as shown in Figure S14b-e. At the initial 0.2 ps, the carrier spatial distribution in both directions is the same, but with time, the profile width in the parallel direction increases sharply, with an expansion rate significantly faster than that in the grating's vertical. The changes of linewidth extracted by Gaussian fitting are shown in Figure S14c, e, which shows a great difference. In the expansion stage, the average speed in the horizontal is 1300 cm^2 s^{-1}, while it is only 800 cm^2 s^{-1} in the vertical. Compared with this, the speed on the uniform substrate is 980 cm^2 s^{-1}. Obviously, the grating not only hinders the hot carrier transport in the vertical but also gives the promotion in the horizontal. In addition, this anisotropy is also reflected in the contraction of the transition region. With the comparison between FigureS14c and e, the contraction amplitude of the vertical is significantly greater than that of the horizontal. Interestingly, in Discussion Section of the text, we attribute the transition's contraction mainly to the speed mismatch between the hot carrier and the exciton, which is appropriate here because the large contraction in the vertical corresponds to the restricted motion of the exciton (the rate of the linear diffusion is nearly zero).

All in all, it can be concluded that the initial expansion of the transient absorption signal observed in the experiment comes from the ultrafast transport of hot carriers in real space. This nonlinear and subsequent cascade transport can be regulated by the substrate.

Note S3. Thermo-optical cascaded carrier transport model

Note S3a: Three-dimensional heat conduction

We simulate the pump-induced carrier and lattice temperature changes in the carrier-lattice-substrate system. As mentioned in the text, the electron and lattice temperatures are calculated by the heat conduction equations as follows:[2,3]

$$C_\mathrm{c}\frac{\partial T_\mathrm{c}}{\partial t} = \nabla\cdot(\boldsymbol{\kappa}_\mathrm{c}\cdot\nabla T_\mathrm{c}) - G(T_\mathrm{c}-T_\mathrm{l}) + S,\ k_\mathrm{cz}\frac{\partial T_\mathrm{c}}{\partial z}|_{z=10\ \mathrm{nm}} = -S_\mathrm{cs} \quad (3a)$$

$$C_\mathrm{l}\frac{\partial T_\mathrm{l}}{\partial t} = \nabla\cdot(\boldsymbol{\kappa}_\mathrm{l}\cdot\nabla T_\mathrm{l}) + G(T_\mathrm{c}-T_\mathrm{l}),\ k_\mathrm{lz}\frac{\partial T_\mathrm{l}}{\partial z}|_{z=10\ \mathrm{nm}} = -S_\mathrm{ls} \quad (3b)$$

$$C_\mathrm{s}\frac{\partial T_s}{\partial t} = \nabla\cdot(k_\mathrm{s}\cdot\nabla T_\mathrm{s}),\ k_\mathrm{s}\frac{\partial T_\mathrm{s}}{\partial z}|_{z=10\ \mathrm{nm}} = S_\mathrm{cs} + S_\mathrm{ls} \quad (3c)$$

where T_c, T_l, and T_s are the temperature of the carrier, lattice, and substrate, respectively. These temperatures are calculated using the finite element method, with the coefficients C_c=2×10^3 J m^{-3} K^{-1} for the carrier heat capacity.[4] C_l=1×10^5 J m^{-3} K^{-1} for the lattice heat capacity.[4] C_s= 6.9×10^5 J m^{-3} K^{-1} for the Si_3N_4 substrate heat capacity.[5] z represents the normal direction, with zero positions at the WS_2 surface and 10 nm at the WS_2-substrate interface. $\boldsymbol{\kappa}_{\mathrm{c(l)}}=\begin{pmatrix} k_{\mathrm{c(l)}} & 0 & 0 \\ 0 & k_{\mathrm{c(l)}} & 0 \\ 0 & 0 & k_{\mathrm{c(l)}z} \end{pmatrix}$ is the thermal conductivity tensor of the WS_2 carrier (lattice), where $k_{\mathrm{c(l)}}$ and $k_{\mathrm{c(l)}z}$ is the in-plane and through-plane thermal conductivity. For in-plane thermal conductivity, $k_\mathrm{c} = k_0\cdot(T_\mathrm{c}/T_\mathrm{l})$, with a simulated k_0=105 W m^{-1} K^{-1}.[6] k_l=31.8 W m^{-1} K^{-1}.[7] While for through-plane thermal conductivity, since the WS_2 thickness is only 10 nm, the specific value of $k_{\mathrm{c(l)}z}$ has little influence on the temperature calculation results. So here, $k_{\mathrm{c(l)}z} \approx k_{\mathrm{c(l)}}/70$.[8] k_s=90 W m^{-1} K^{-1} is the Si_3N_4 thermal conductivity.[5] G=2×10^{15} W

m^{-3} K^{-1} is the carrier-phonon coupling rate.[9] S_{cs} (S_{ls}) is the interfacial heat exchange rate between the carrier (lattice) and substrate, both set to be 1 MW m^{-2} K^{-1}.[10,11] $S(\boldsymbol{r},t)$ is the initial heat source depending on the pump pulse, which can be defined as[12]

$$S = \frac{2AF\sqrt{\ln 2}}{d\cdot\tau_p\sqrt{\pi}}\cdot exp\left[-\frac{z}{d}-\frac{x^2+y^2}{2\sigma_{\mathrm{p}}^2}-\frac{4\ln 2(t-2\tau_{\mathrm{p}})^2}{\tau_{\mathrm{p}}^2}\right] \tag{4}$$

where A~0.1 is pump absorbance at a photon energy of 3.1 eV, F is the pump fluence varying from 8 to 80 μJ cm^{-2}, with an FWHM duration of τ_{p}=200 fs and spot width of σ_{p}=0.9 μm. The penetration depth d can be expected to be 10 nm according to an absorption coefficient of 1×10^{8} m^{-1}.[13,14]

For two limiting cases of the cascaded carrier transport, the initial hot carrier expansion rate can be roughly estimated to be $D_1 = k_{\mathrm{c}}/C_{\mathrm{c}} \propto T_{\mathrm{c}}/T_{\mathrm{l}}$. Upon pump excitation, T_{l} stays almost the same, only increased by ~20 K, while T_{c} drastically increase to thousands of Kelvin within 1 ps. Thus the fast expansion rate is roughly proportional to T_{c}, which is well consistent with previous reports in semiconductor silicon.[15] In contrast, the long-term exciton diffusion is governed by the lattice thermal transport $D_2 = (k_{\mathrm{l}} + k_{\mathrm{c}})/(C_{\mathrm{c}}+C_{\mathrm{l}}) \approx (k_{\mathrm{l}} + k_{\mathrm{c}})/C_{\mathrm{l}}$, which is approximately constant at different time delays due to the small change of T_{c} and T_{l} after 2 ps.[4]

Note S3b: Permittivity calculation

The temperature change is further reflected onto $\Delta R/R_0$ through thermo-optical conversion described below. Specifically, the photo-induced complex permittivity (ε) can be attributed to the free carrier (hot carrier) and bound exciton species, which is described using a Drude-Smith and Lorentz model, respectively.[16,17]

$$\varepsilon = \varepsilon_\infty + \frac{f_{\mathrm{ex}}}{E_{\mathrm{ex}}^2-E_{\mathrm{probe}}^2-i\gamma E_{\mathrm{probe}}} + \frac{iD_0}{\omega_{\mathrm{probe}}(1-i\omega_{\mathrm{probe}}\tau)(1-\frac{C}{1-i\omega_{\mathrm{probe}}\tau})} \tag{5}$$

where ε_∞=15 is the fitted high-frequency contribution to the real part of ε. The second term represents the A-exciton Lorentzian contributions, where f_{ex}=4.2 is the fitted oscillator

strength at resonance energy of E_{ex}=1.98 eV, and E_{probe}=2.06 eV is the probe photon energy. $\gamma = \gamma_0 \cdot (T_l/T_0)$ is the A-exciton broadening extracted from the linear absorption spectrum, which is proportional to the lattice temperature T_l, with room temperature T_0=293 K and corresponding broadening γ_0=35 meV.[17,18] The third term describes the Drude-Smith contributions from free carriers (hot carriers), where D_0=5·10^{-6}·w is the Drude weight proportional to the density ratio (w) between the hot carrier (N_{hot}) and exciton (N_{ex}).[16] It can be roughly estimated as $w = \frac{N_{\mathrm{hot}}}{N_{\mathrm{ex}}} \approx \frac{1}{\exp(\frac{E_{\mathrm{b}}}{k_{\mathrm{B}}T_{\mathrm{c}}})-1}$ under the assumption that thermal equilibrium is approached between the two excited states,[19] with an exciton binding energy of $E_{\mathrm{b}}\sim$50 meV[20] for a 10 nm layered WS_2 and Boltzmann constant k_B. ω_{probe} is the probe angular frequency. C $(0 \le C \le 1)$ is the coefficient determining the degree of carrier localization, with a near-zero value indicating a tendency to the Drude free-carrier limit, here C is set as 0.85.[16] $\tau = 10^{-10} \cdot T_{\mathrm{c}}^{-1.5}$ is the carrier-carrier scattering time[21] that depends on carrier temperature, as discussed in the main text.

Note S3c: Acquisition of differential reflection signal

Finally, the photo-induced complex permittivity is transferred to optical reflectivity through Fresnel equation in a thin-film (air-WS_2-Si_3N_4-Ag) under normal incidence

$$R = \left|\frac{r_1 e^{i\phi_1+i\phi_2}+r_2 e^{i\phi_2-i\phi_1}+r_3 e^{-i\phi_1-i\phi_2}+r_1 r_2 r_3 e^{i\phi_1-i\phi_2}}{e^{i\phi_1+i\phi_2}+r_1 r_2 e^{i\phi_2-i\phi_1}+r_1 r_3 e^{-i\phi_1-i\phi_2}+r_2 r_3 e^{i\phi_1-i\phi_2}}\right|^2 \quad (6)$$

where $r_1 = \frac{n_{\mathrm{air}}-\sqrt{\varepsilon}}{n_{\mathrm{air}}+\sqrt{\varepsilon}}$, $r_2 = \frac{\sqrt{\varepsilon}-n_{\mathrm{Si_3N_4}}}{\sqrt{\varepsilon}+n_{\mathrm{Si_3N_4}}}$, $r_3 = \frac{n_{\mathrm{Si_3N_4}}-n_{\mathrm{Ag}}}{n_{\mathrm{Si_3N_4}}+n_{\mathrm{Ag}}}$ are the reflectivity at air-WS_2, WS_2-Si_3N_4, Si_3N_4-Ag interfaces, respectively, with the refractive index of air n_{air}=1, Si_3N_4 thin film $n_{\mathrm{Si_3N_4}}$= 2 and Ag substrate n_{Ag}=0.057+4i for 2.06 eV probe.[22] $\phi_1 = \frac{2\pi d_{\mathrm{WS_2}}\mathrm{Re}(\sqrt{\varepsilon})}{\lambda_{\mathrm{probe}}}$ and $\phi_2 = \frac{2\pi d_{\mathrm{Si_3N_4}}\mathrm{Re}(n_{\mathrm{Si_3N_4}})}{\lambda_{\mathrm{probe}}}$ represent the optical path increments of the probe beam passing through WS_2 and Si_3N_4, with thicknesses of $d_{\mathrm{WS_2}}$=10 nm and $d_{\mathrm{Si_3N_4}}$=180 nm and probe wavelength of 603 nm.

As a result, the transient differential reflectivity at specific time delays (t) and in-plane positions (x, y) can be expressed as

$$\frac{\Delta R(x,y,t)}{R_0} = \frac{R(x,y,t)-R_0}{R_0} \quad (7)$$

where R_0 represents the reflectivity of the sample before excitation at room temperature.

In our measurement time range, carrier-carrier scattering leads to the carrier thermalization and a drastic rising of the carrier temperature, resulting in a super-expansion of the hot carrier in the first ~1 ps. After that, carrier-phonon coupling leads to the decrease of carrier temperature and the formation of bound exciton at ~2 ps. Finally, the long-term excited state diffusion is dominated by the slow exciton diffusion, which is limited by the lattice thermal transport. By convolving them with the probe pulse and performing Gaussian fitting at different delay times, we can directly calculate the transport dynamics of the excited states and thus reproduce the experimental results.

Note S4. Cascaded carrier transport probed at the PB and low-energy PA resonance.

We note that the carrier diffusion dynamics probed at exciton resonance (PB feature at 1.98 eV) and low-energy PA resonance (1.92 eV) are completely different from that at high-energy PA feature (2.06 eV), as shown in Figure S8. According to the discussion in the main text, the high-energy PA feature is attributed to the hot carriers, while the PB and low-energy PA features are primarily caused by the band-edge bound excitons. Below, we will give a detailed discussion on the deviation of diffusion dynamics of the three features.

We firstly look at the spatial evolution of the PB feature, which represents the diffusion of the bound exciton. As shown in Figure S8a, the bound exciton exhibits a smaller initial expansion length (310 nm) at the first 1.4 ps and a larger contraction length (-150 nm) at the transition region, as compared to that of the imaging of 2.06 eV PA (500 nm expansion and -50 nm contraction in Figure 2e). This deviation can be explained as follows: First, at the

initial 1.4 ps, the expansion behavior of bound exciton is not caused by the intrinsic diffusion of the excitons generated at the pump center, but by the local cooling of the expanded hot carriers. Thus, the initial expansion of the exciton is slowed down compared with the hot carrier due to the delay effect of electron-phonon coupling. Second, at the transition region (1.4~2 ps), three effects may cause a larger contraction for the bound excitons: 1. Slightly different mobility of electrons and holes, which separates them in real space. 2. Carriers with a specific polarity (electrons or holes) are trapped by the defect states (WS_2 is widely considered as an *n*-type semiconductor due to sulfur vacancy, which tends to capture holes). Based on these two effects, the density ratio of hot carriers to excitons increases with the diffusion length, leading to a larger contraction of the exciton diffusion at the transition region. 3. It can be noticed that free carriers which also contribute to PB initially are more efficient on inducing transient absorption than excitons. [23] Then, the free carriers are converted into excitons accompanied by the signal's fast decay, during which the initial free carriers could produce large expansion transport, while excitons will not. These free-carrier spatiotemporal dynamics lead to the ultrafast expansion of PB signal, but then decline the PB generated by those diffused carriers as well. Due to the retention of substantial excitons at the center, the proportion between central and surrounding PB will increase, causing a concentration towards the center.

The low-energy PA feature peaks at 1.92 eV, ~60 meV lower than the exciton resonance. This feature exhibits roughly the same kinetics as that of the PB feature (Figure S4), indicating that it should be originated from the bound exciton. Generally, this low-energy feature may be caused by two effects: biexciton absorption[24] or trapped-state exciton formation. Here we believe that biexciton formation is the dominant mechanism due to the diffusion dynamics shown in Figure S8b. Specifically, since the exciton density at the pump center is much higher than that at surroundings, biexciton generation efficiency is much higher at the pump center, leading to an initial contraction of the PA feature. In contrast, the

center's high exciton density will saturate the local defect state, resulting in a smaller probe absorption compared to that at surroundings, and thus an initial expansion rather than contraction occurs for the PA feature. According to the experimental results in Figure S8b, an initial contraction with D_1=-240 cm^2 s^{-1} is found, indicating that the PA feature at 1.92 eV is caused by the generation of biexcitons. The 60 meV energy offset is roughly the same as the biexciton binding energy,[25] further supporting this ascription.

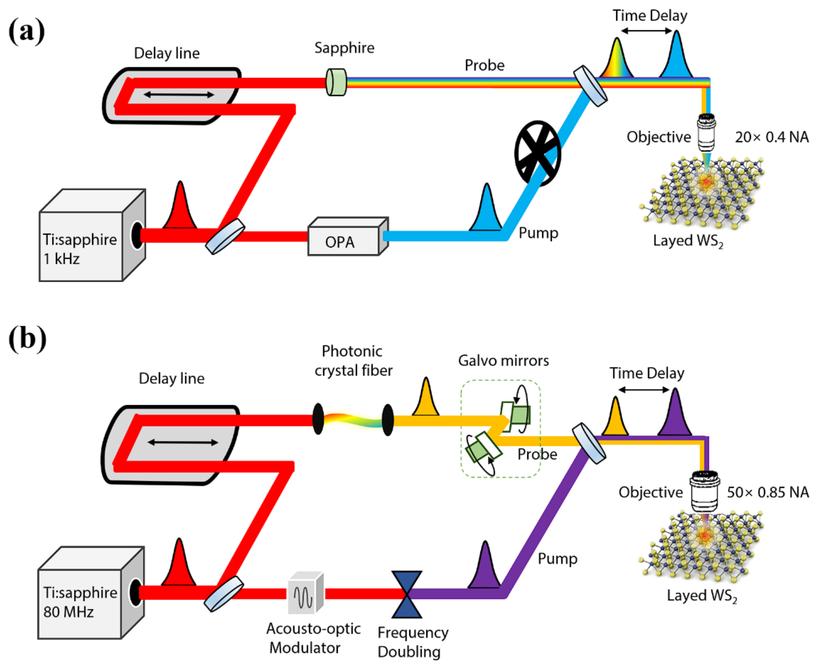

Figure S1. Schematic of pump-probe techniques. a) Schematic system for the femtosecond pump-probe spectroscopy, providing the temporal evolution of the $\Delta R/R_0$ spectrum. b) Schematic of the spatiotemporal TAM, which provides spatiotemporal imaging of the carrier distribution (asynchronous scanning mode) and confocal imaging of the sample morphology (synchronous scanning).

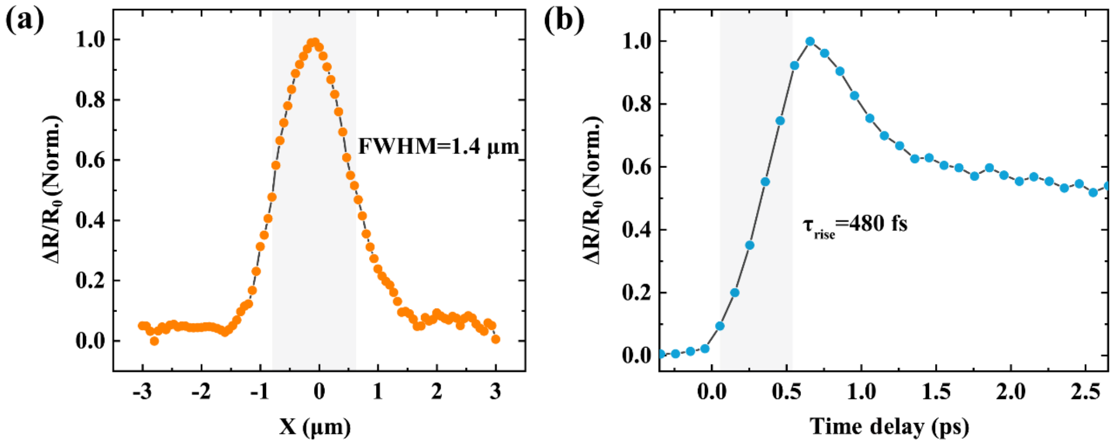

Figure S2. Determination of spatiotemporal resolution in TAM. a) Spatial resolution represented by FWHM=1.4 μm. b) Rising edge from 10% to 90% maximum with τ_{rise}=480 fs. The pump and probe wavelengths are 400 and 620 nm, respectively. These two values are obtained by testing a defective monolayer CVD-growth WS_2 sample in which the carrier transport is limited and the temporal dynamics are faster than multilayers. Even so, here the actual resolution of the system will be better than the measured value which is a convolution between that and the carrier dynamic.

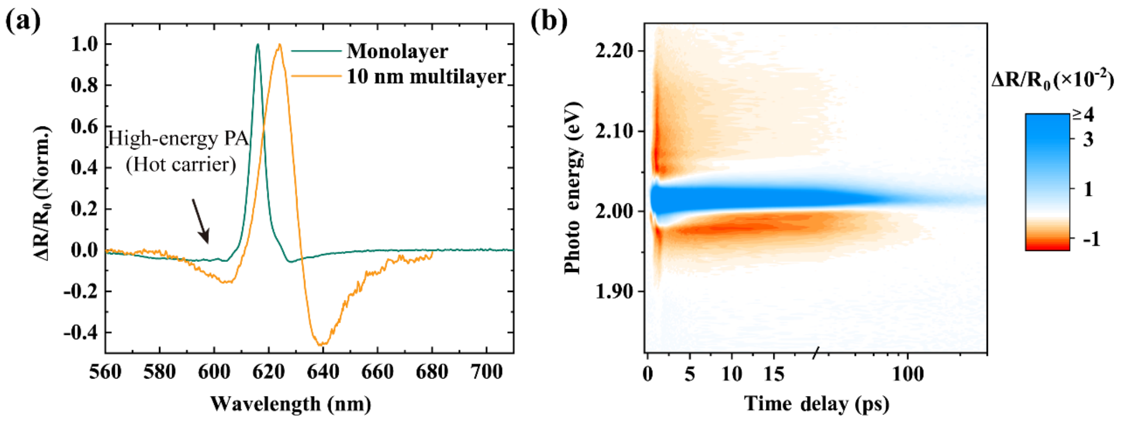

Figure S3. Typical $\Delta R/R_0$ spectra of WS_2. a) Transient $\Delta R/R_0$ traces of monolayer and multilayer WS_2 at the maximum signal delay. b) Compared with multilayer WS_2 (Figure 2a) in the main text, the A-exciton resonance (the PB feature, blue area) is blue-shift and exhibits faster decay dynamics. The two PA features on both sides of the PB feature are also found here, consistent with the multilayer sample.

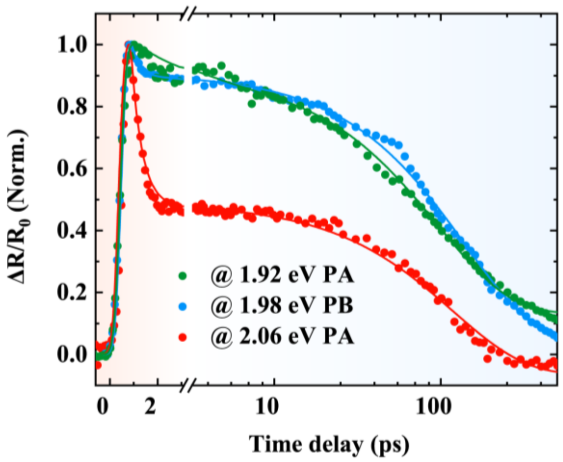

Figure S4. Long-term hot carrier and excitonic temporal dynamics. The PB and 1.92 eV PA features show roughly the same kinetics, with a tiny fast relaxation (τ_{fast}=0.3 ps, A_{fast}=10% for PB, and τ_{fast}=2.1 ps, A_{fast}=10% for 1.92 eV PA) followed by a dominant slow decay (τ_{slow}=140 ps, A_{slow}=90% for PB, and τ_{slow}=100 ps, A_{slow}=90% for 1.92 eV PA). While the 2.06 eV PA feature exhibits a distinct dynamic, with comparable fast (τ_{fast}=0.5 ps, A_{fast}=51%) and slow (τ_{slow}=120 ps, A_{slow}=49%) decays.

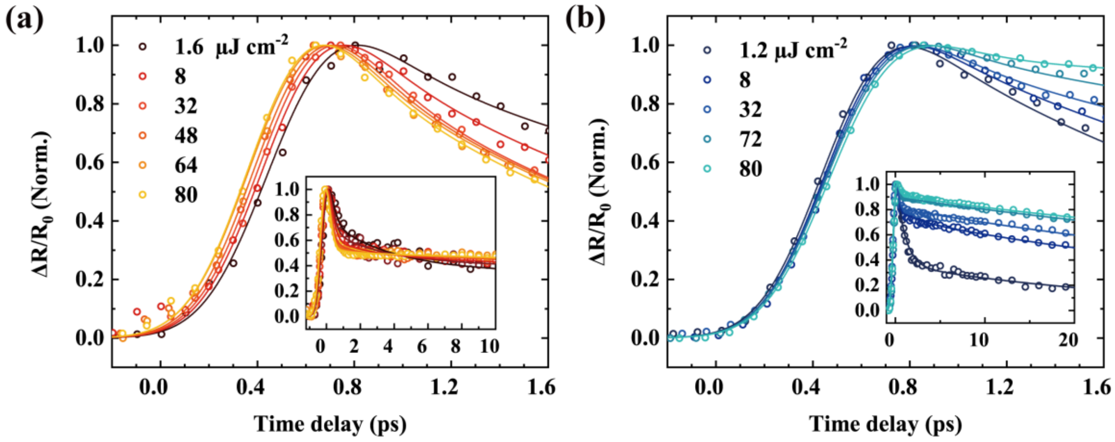

Figure S5. Fluence-dependent dynamics for build-up processes. a) The ultrafast build-up process of the hot carriers (2.06 eV PA) at different pump fluences. Inset: The corresponding long-term carrier relaxation. All the curves are normalized to highlight the peaking times, with the open circles (lines) representing the experimental results (exponential fittings). b) the same to (a), but probed at exciton resonance (1.98 eV PB). With increasing pump fluence, the build-up process speeds up for hot carriers due to a promotion of the carrier-carrier scattering, while slowing down for band-edge exciton due to the phonon bottleneck effect.

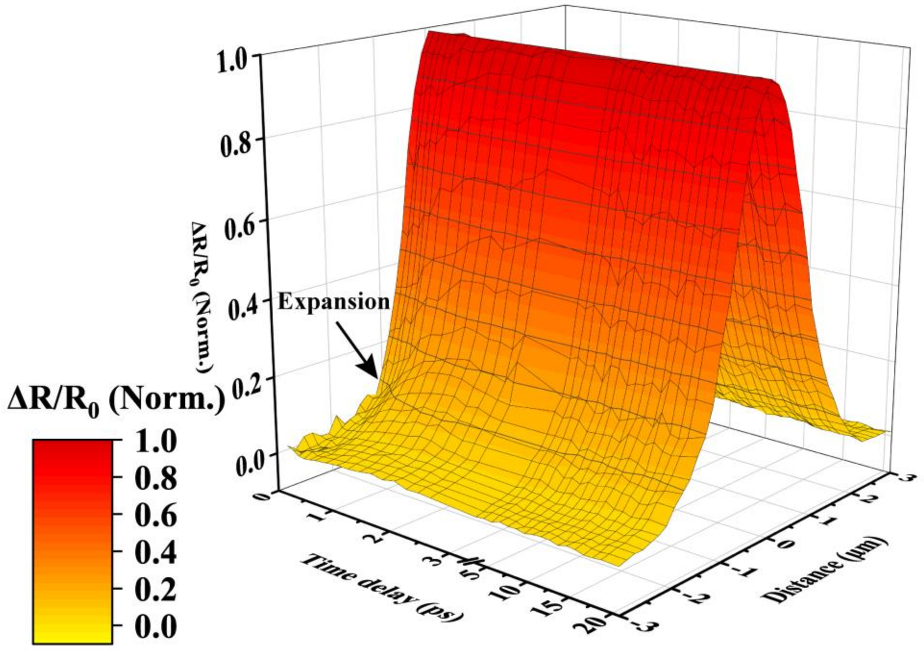

Figure S6. Complete one-dimensional normalized $\Delta R/R_0$ imaging recorded within 20 ps. With a 3.1 eV pump (80 μJ cm^{-2}) and 2.06 eV probe, the initial uplift (before ~1.4 ps) can be clearly observed corresponding to the hot-carrier expansion in Figure 2c, e, where the subsequent contraction and linear diffusion can be obtained by Gaussian fitting.

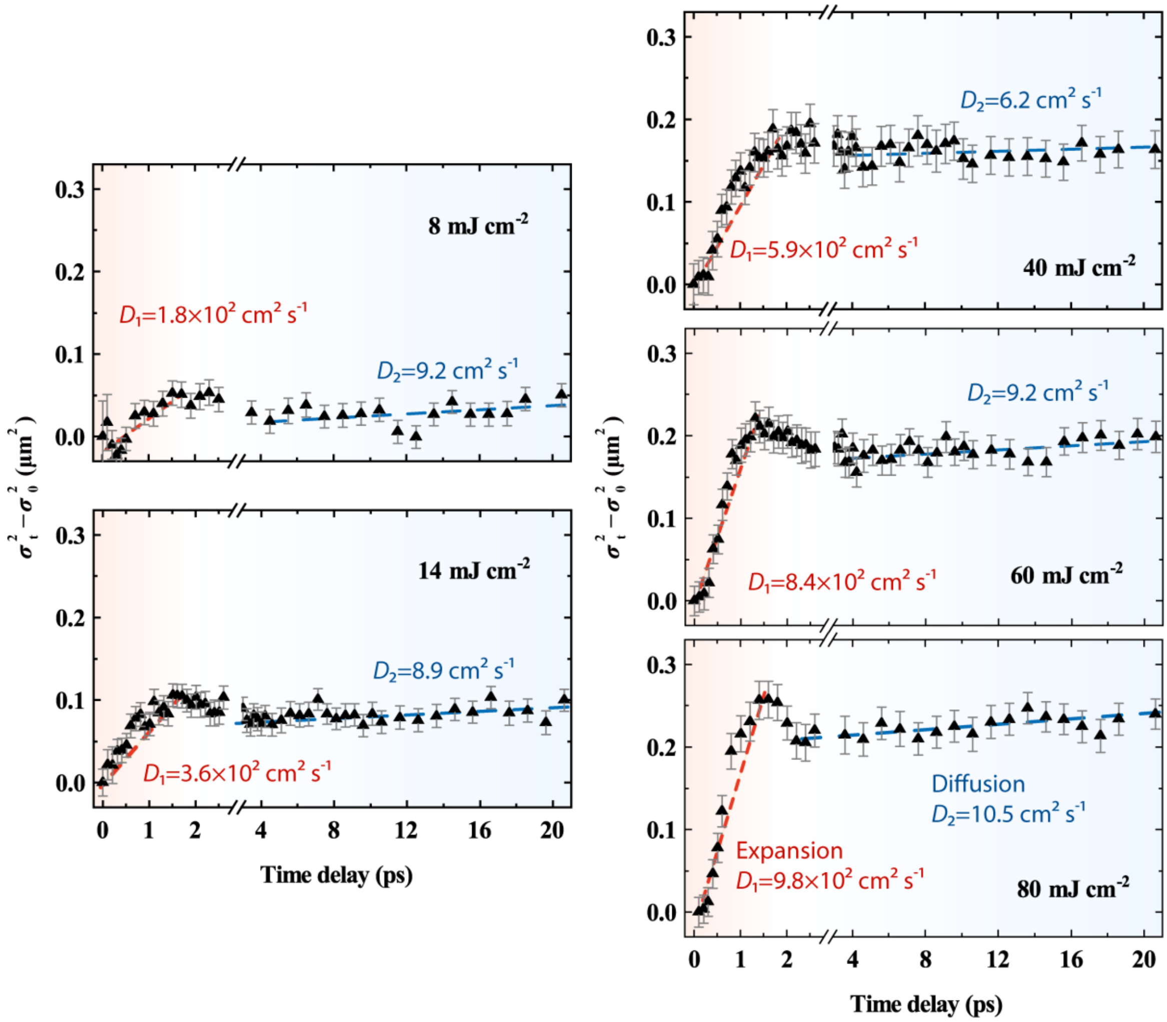

Figure S7. Fluence-dependent diffusion dynamics. With increasing pump fluence, the hot carrier expansion rate (D_1) increases due to the drastic rise of the carrier temperature (T_c), while the slow exciton diffusion rate (D_2) roughly keeps constant because there is only a slight rise in the lattice temperature (T_l).

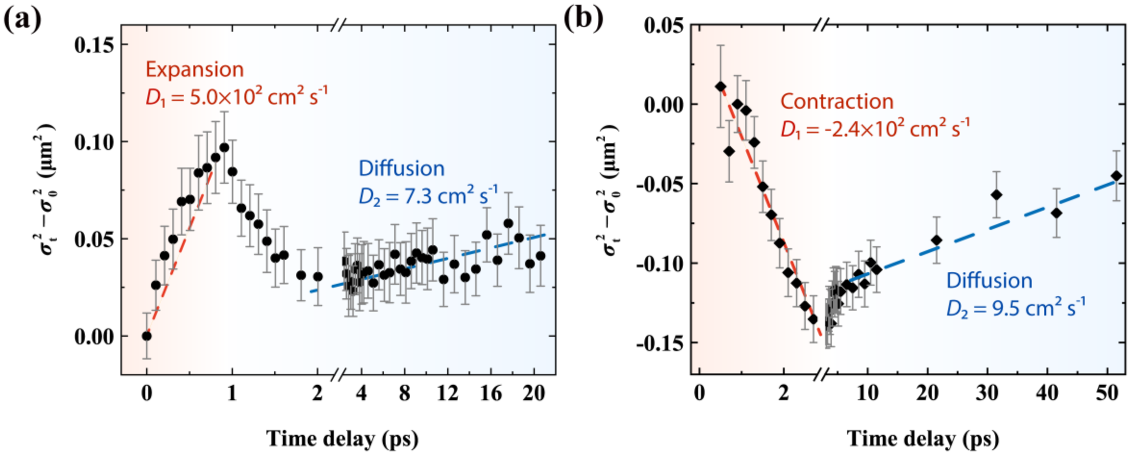

Figure S8. Diffusion dynamics of excitonic species. a) Diffusion dynamics probed at 1.98 eV for excitons. b) Same as (a) but probed at 1.92 eV for biexcitons. The dashed lines are used for guides only.

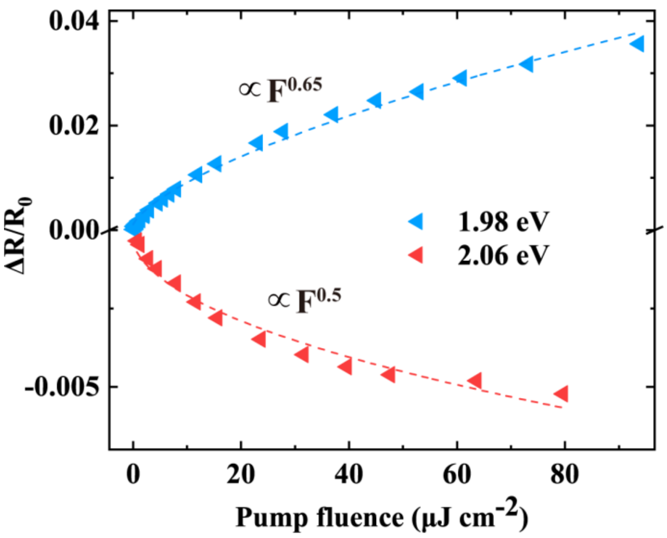

Figure S9. Extracted fluence-dependent peak values of $\Delta R/R_0$. Dots are experimental data of the high-energy PA (2.06 eV) and PB (1.98 eV) features, with the density dependence that is exponentially fitted by the dashed curves. The two follow the exponential growth of 0.5 and 0.65, respectively.

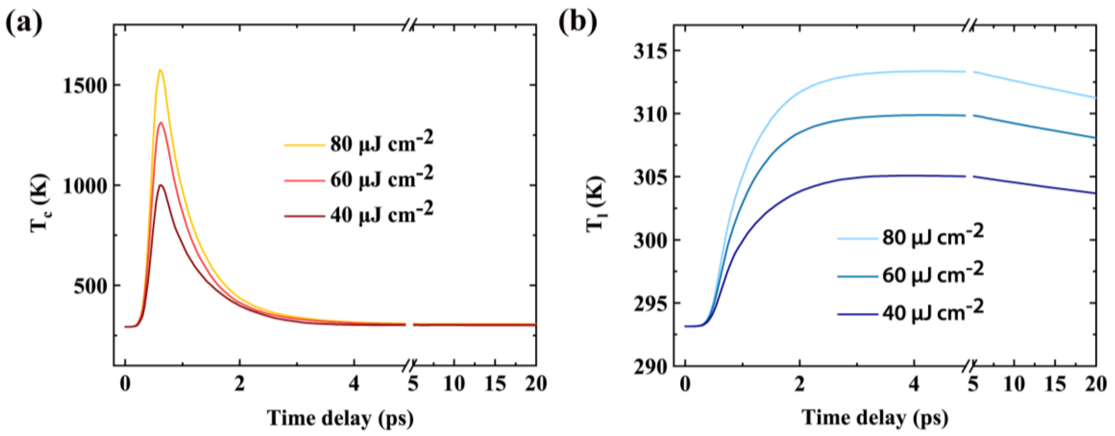

Figure S10. Calculated temperature dynamics. a) Dynamics of carrier temperature at $x = y = z = 0$ under different pump fluences. b) Same as (a) for lattice temperature.

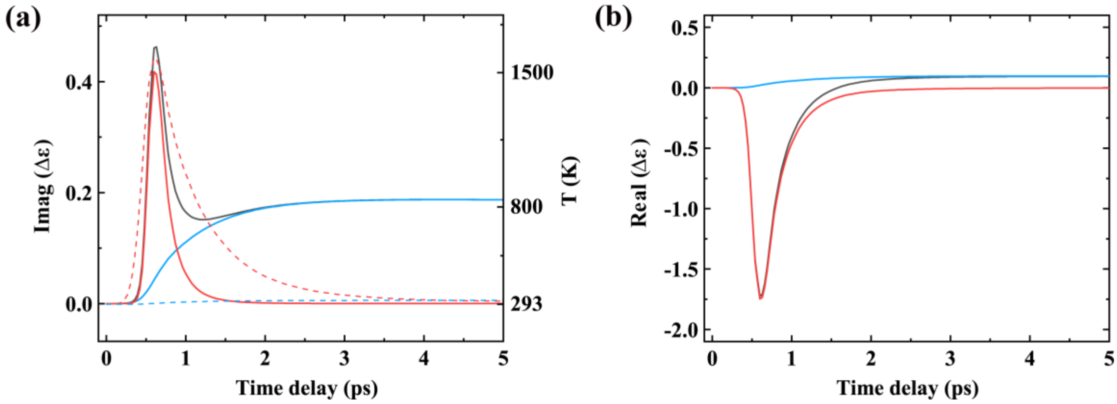

Figure S11. Hot carrier and excitonic contributions to the complex permittivity change. a,b)The imaginary (a) and real (b) parts of the permittivity change induced by hot carriers (red solid), bound excitons (blue solid), and their combination (black solid). The dashed lines in (a) are the calculated carrier (red dash) and lattice (blue dash) temperatures for reference.

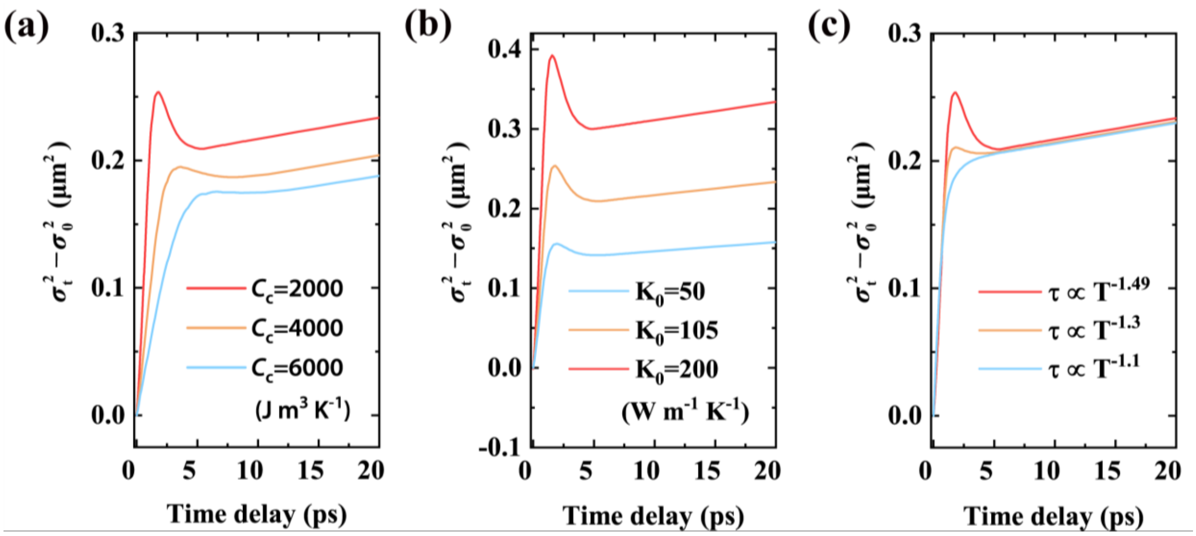

Figure S12. Predicted modulation of hot carrier expansion in cascaded transport model. a,b) Modulation for $\sigma_t^2 - \sigma_{t_0}^2$ through the change of carrier heat capacity (a), and that of carrier thermal conductivity is in (b). c) Modulation from carrier-carrier scattering time (τ) tuned by carrier temperature via $\tau \propto T_c^{-\alpha}$, a larger negative exponent means less contribution of the hot carrier to $\Delta R/R_0$ and weakened expansion scale.

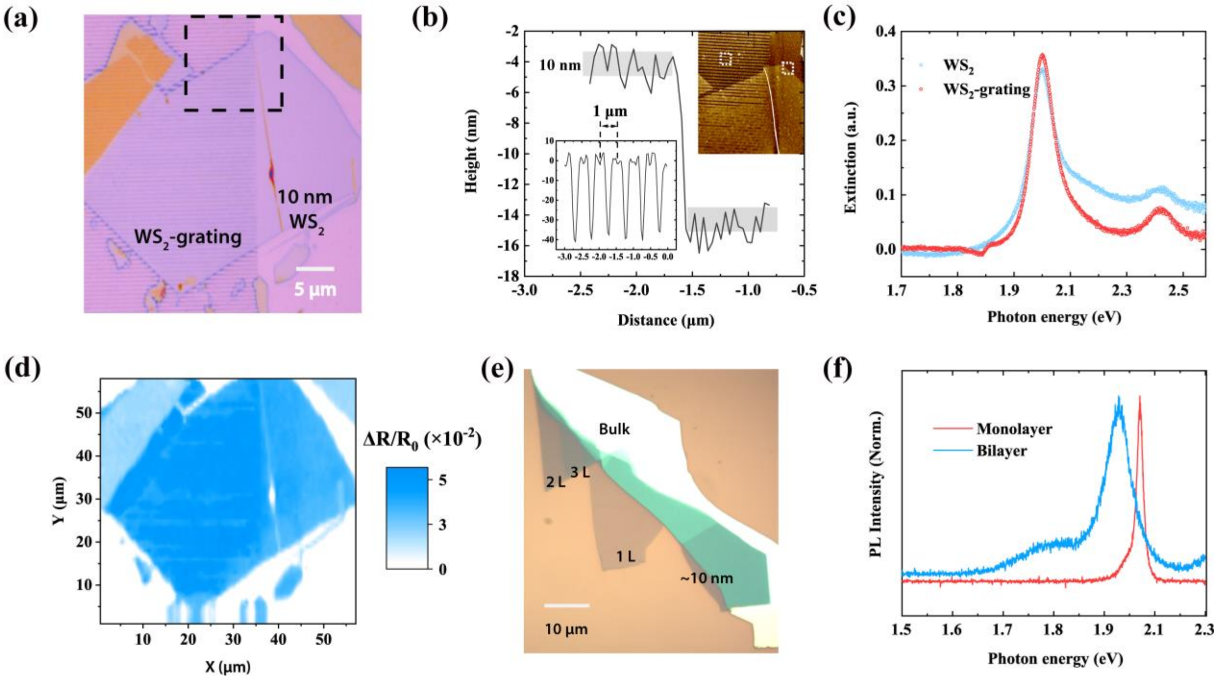

Figure S13. Sample characterization. a) Optical image of the transferred WS_2 on a partially etched Si_3N_4-Ag substrate. The dashed box is the scanning area of atomic force microscopy (AFM). b) AFM height profile and image (inset) of the sample in (a), showing a ~10 nm WS_2 thickness and a period of 1 μm grating structure at the two dashed positions. c) Linear reflection spectra of WS_2 and WS_2-grating. The WS_2-grating maintains the spectral position of the A- exciton, but with a stronger extinction. d) Morphology image of the sample in (a) obtained by 2D synchronous scanning of TAM. e) Optical image of the exfoliated WS_2 on Si/SiO_2 substrate with marked thickness. f) Photoluminescence of monolayer and bilayer WS_2 in (e).

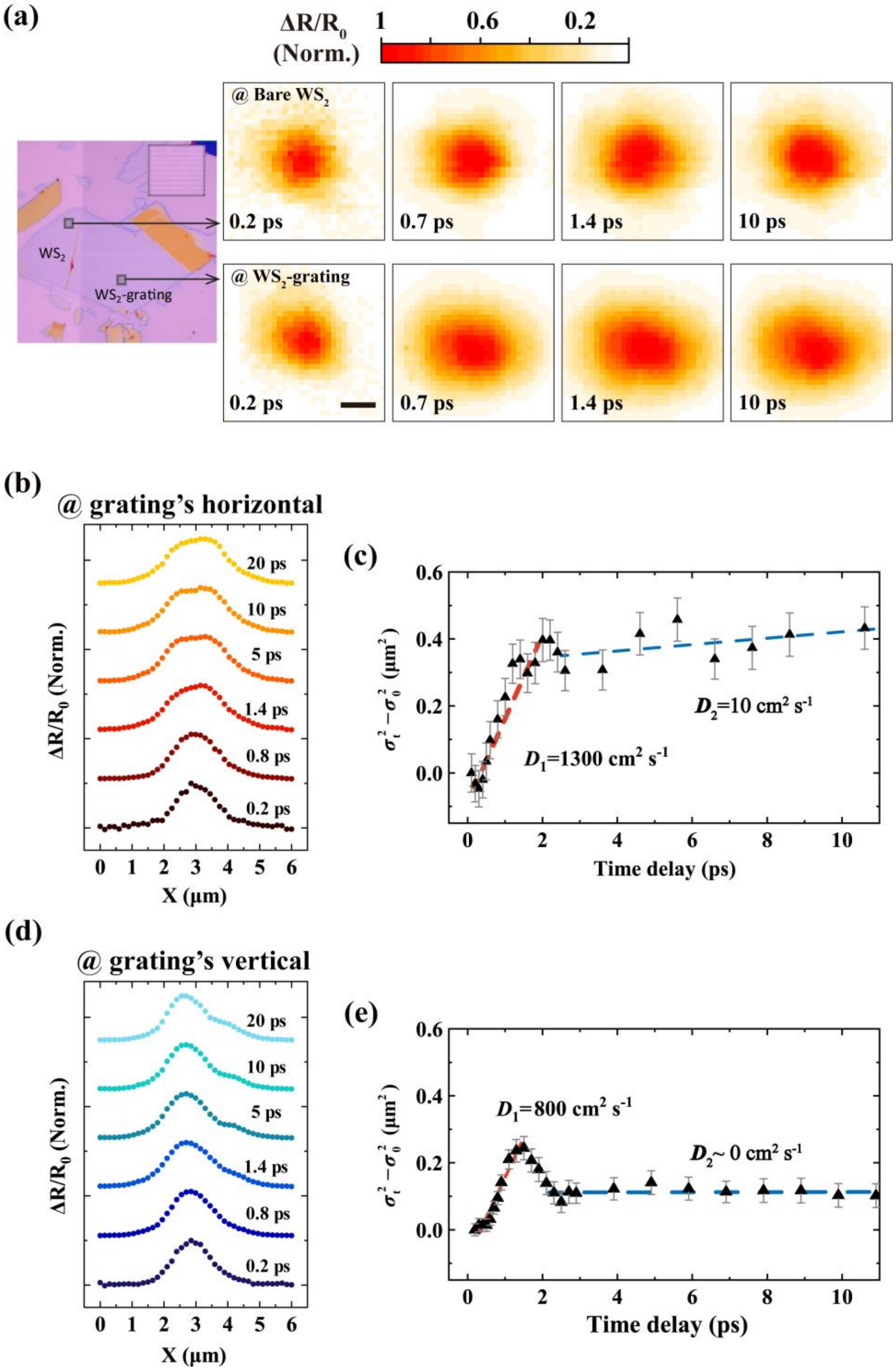

Figure S14. Imaging of anisotropic transport on the grating structure. a) 2D images of bare WS_2 and WS_2-grating at different delay times, the probe is 2.06 eV. All the figures are normalized to highlight the transport dynamics. Drastic hot carrier expansion is observed in the first 1.4 ps, which is more significant for WS_2-grating along grating's horizontal with anisotropic transport. Scale bar: 1 μm. b-e) 1D images of 2.06 eV probe along the grating's horizontal (b) and vertical (d) at different delay times, with fitting results shown in (c, e).

Reference

[1] Z. Guo, J. S. Manser, Y. Wan, P. V. Kamat, L. Huang, *Nat. Commun.* **2015**, *6*, 7471.
[2] A. Block, M. Liebel, R. Yu, M. Spector, Y. Sivan, F. J. García de Abajo, N. F. van Hulst, *Sci. Adv.* **2019**, *5*, eaav8965.
[3] R. H. M. Groeneveld, R. Sprik, A. Lagendijk, *Phys. Rev. B* **1995**, *51*, 11433.
[4] E. J. Sie, A. Steinhoff, C. Gies, C. H. Lui, Q. Ma, M. Rösner, G. Schönhoff, F. Jahnke, T. O. Wehling, Y. H. Lee, J. Kong, P. Jarillo-Herrero, N. Gedik, *Nano Lett.* **2017**, *17*, 4210.
[5] H. S. Dow, W. S. Kim, J. W. Lee, *AIP Adv.* **2017**, *7*, 095022.
[6] A. N. Gandi, U. Schwingenschlögl, *Chem. Mater.* **2014**, *26*, 6628.
[7] B. Peng, H. Zhang, H. Shao, Y. Xu, X. Zhang, H. Zhu, *RSC Adv.* **2016**, *6*, 5767.
[8] A. Pisoni, J. Jacimovic, R. Gaál, B. Náfrádi, H. Berger, Z. Révay, L. Forró, *Scripta Materialia* **2016**, *114*, 48.
[9] R. Wang, H. Zobeiri, Y. Xie, X. Wang, X. Zhang, Y. Yue, *Adv. Sci.* **2020**, *7*, 2000097.
[10] K. Wei, Y. Sui, Z. Xu, Y. Kang, J. You, Y. Tang, H. Li, Y. Ma, H. Ouyang, X. Zheng, X. Cheng, T. Jiang, *Nat. Commun.* **2020**, *11*, 3876.
[11] E. Yalon, Ö. B. Aslan, K. K. H. Smithe, C. J. McClellan, S. V. Suryavanshi, F. Xiong, A. Sood, C. M. Neumann, X. Xu, K. E. Goodson, T. F. Heinz, E. Pop, *ACS Appl. Mater. Interfaces* **2017**, *9*, 43013.
[12] J. Zhang, Y. Chen, M. Hu, X. Chen, *J. Appl. Phys.* **2015**, *117*, 063104.
[13] W. Zhao, Z. Ghorannevis, L. Chu, M. Toh, C. Kloc, P.-H. Tan, G. Eda, *ACS Nano* **2013**, *7*, 791.
[14] A. R. Beal, W. Y. Liang, H. P. Hughes, *J. Phys. C: Solid State Phys.* **1976**, *9*, 2449.
[15] E. Najafi, V. Ivanov, A. Zewail, M. Bernardi, *Nat. Commun.* **2017**, *8*, 15177.
[16] C. He, L. Zhu, Q. Zhao, Y. Huang, Z. Yao, W. Du, Y. He, S. Zhang, X. Xu, *Adv. Opt. Mater.* **2018**, *6*, 1800290.
[17] Y. Jiang, H. Wang, S. Wen, H. Chen, S. Deng, *ACS Nano* **2020**, *14*, 13841.
[18] C. Ruppert, A. Chernikov, H. M. Hill, A. F. Rigosi, T. F. Heinz, *Nano Lett.* **2017**, *17*, 644.
[19] A. Steinhoff, M. Florian, M. Rösner, G. Schönhoff, T. O. Wehling, F. Jahnke, *Nat. Commun.* **2017**, *8*, 1166.
[20] A. Chernikov, T. C. Berkelbach, H. M. Hill, A. Rigosi, Y. Li, O. B. Aslan, D. R. Reichman, M. S. Hybertsen, T. F. Heinz, *Phys. Rev. Lett.* **2014**, *113*, 076802.
[21] Z. Nie, R. Long, L. Sun, C.-C. Huang, J. Zhang, Q. Xiong, D. W. Hewak, Z. Shen, O. V. Prezhdo, Z.-H. Loh, *ACS Nano* **2014**, *8*, 10931.
[22] P. B. Johnson, R. W. Christy, *Phys. Rev. B* **1972**, *6*, 4370.
[23] E. J. Sie, A. J. Frenzel, Y.-H. Lee, J. Kong, N. Gedik, *Phys. Rev. B* **2015**, *92*, 125417.
[24] D. K. Zhang, D. W. Kidd, K. Varga, *Nano Lett.* **2015**, *15*, 7002.
